# To meet grand challenges, agricultural scientists must engage in the politics of constructive collective action

**DOI:** 10.1002/csc2.20318

**Published:** 2020-11-22

**Authors:** N. Jordan, J. Gutknecht, K. A. Bybee‐Finley, M. Hunter, T. J. Krupnik, C. M. Pittelkow, P. V. V. Prasad, S. Snapp

**Affiliations:** ^1^ University of Minnesota 1991 Upper Buford Circle St. Paul MN 55108 USA; ^2^ University of Minnesota 1529 Gortner Avenue St. Paul MN 55108 USA; ^3^ Cornell University 519 Bradfield Hall Ithaca NY 14853 USA; ^4^ American Farmland Trust 1991 Upper Buford Circle St. Paul MN 55108 USA; ^5^ International Maize and Wheat Improvement Center (CIMMYT) House 10/B. Road 53. Gulshan‐2 Dhaka 1213 Bangladesh; ^6^ University of California, Davis One Shields Avenue Davis CA 95616 USA; ^7^ Kansas State University 108 Waters Hall, 1603 Old Claflin Place Manhattan KS 66506 USA; ^8^ Michigan State University Plant and Soil Sciences Building, 1066 Bogue St., Room 490 East Lansing MI 48824 USA

## Abstract

Agriculture now faces grand challenges, with crucial implications for the global future. These include the need to increase production of nutrient‐dense food, to improve agriculture's effects on soil, water, wildlife, and climate, and to enhance equity and justice in food and agricultural systems. We argue that certain politics of constructive collective action—and integral involvement of agricultural scientists in these politics—are essential for meeting grand challenges and other complex problems facing agriculture in the 21st century. To spur reflection and deliberation about the role of politics in the work of agricultural scientists, we outline these politics of constructive collective action. These serve to organize forceful responses to grand challenges through coordinated and cooperative action taken by multiple sectors of society. In essence, these politics entail (1) building bonds of affinity within a heterogenous network, (2) developing a shared roadmap for collective action, and (3) taking sustained action together. These emerging politics differ markedly from more commonly discussed forms of political activity by scientists, e.g., policy advisory, policy advocacy, and protest. We present key premises for our thesis, and then describe and discuss a politics of constructive collective action, the necessary roles of agricultural scientists, and an agenda for exploring and expanding their engagement in these politics.

## 1 INTRODUCTION

We argue that certain politics of constructive collective action—and the integral involvement of agricultural scientists in these politics—are essential for meeting grand challenges facing agriculture in the 21st century. Broadly, these are to increase production of nutrient‐dense food (Willett et al., 2019), to improve agriculture's effects on soil, water, wildlife, and climate (Rockström et al., 2017), and to enhance equity and justice in food and agricultural systems across scales (Loos et al., 2014). Present rates of progress on these challenges are mixed at best (Steiner et al., 2020). All represent urgent and complex (or “wicked”) problems, defined as (i) multidimensional, (ii) highly uncertain and unpredictable and (iii) involving multiple stakeholder groups that do not have a common understanding of the problem and potential solutions (Levin, Cashore, Bernstein, & Auld, 2012). The politics of constructive collective action, outlined below, organize forceful responses to such problems through coordinated and cooperative action taken by multiple sectors of society. These emerging politics differ markedly from more commonly discussed forms of political activity by scientists, e.g., advising governments on policy, advocacy for policies, and protest. We argue that participation in politics of constructive collective action is necessary to fully engage the power of agricultural science in meeting urgent and complex problems. We first present key premises for our thesis, and then define these politics, the necessary roles of agricultural scientists, and an agenda for expanding the engagement of scientists. Our key message is that agricultural scientists can greatly heighten their contributions to meeting complex problems if they understand and participate—as scientists—in these particular politics. Yet, to be highly effective participants, they must recognize, embrace, and build skills for a new identity and role: scientist as *skilled actor in the constructive politics of collective action*.

## 2 KEY PREMISES

### 2.1 Premise I: A broad sweep of innovation is underway in response to agriculture's grand challenges

In response to these challenges, many relevant innovations are emerging. Some are technological (Herrero et al., 2020), while others are economic, political, or cultural in nature (Leeuwis & Aarts, 2011). Examples include new crops and cropping practices for sustainable intensification of crop production, e.g., perennial crops (Glover et al., 2010), gene editing (Ma, Mau, & Sharbel, 2018), participatory breeding (Weltzien & Christinck, 2017), cooperative and integrated pest management (Evans et al., 2018), and novel cooperative food hub market systems that integrate across small‐scale production units (Cleveland, Müller, Tranovich, Mazaroli, & Hinson, 2014). Complementary innovations are also emerging for creating value from these new crops and practices, e.g., novel bio‐refining methods for producing products from crops (Chen & Zhang, 2015), agro‐informatics (Delgado, Short, Roberts, & Vandenberg, 2019), conservation finance (Whelpton & Ferri, 2017), responsible innovation methods that seek “social license” to advance agricultural technologies (Kuzma, 2019), and governance systems that integrate “top‐down” and “bottom‐up” strategies for advancing new crops and practices (Dorsch & Flachsland, 2017).

### 2.2 Premise II: To meet agriculture's grand challenges, innovation must be integrated and leveraged by collective action

To realize the full potential of current innovation, certain forms of collective action—coordinated and strategic actions taken by a broad range of societal sectors, such as farmers, agribusinesses, governments, and advocacy groups—are essential. First, collective action is needed to integrate new technologies with other, complementary innovations to create novel socio‐technical systems for agriculture (Leeuwis & Aarts, 2011). Such systems are integrated sets of technical and social innovations that function together to meet a societal need related to agriculture (Meynard et al., 2018). After creation of novel systems, further and sustained collective action is needed to refine, adapt, and scale these novel systems (Wigboldus et al., 2016) in response to the shifting nature of complex problems.

**Core Ideas**

- Meeting agriculture's grand challenges requires coordinated technical and social innovation
- Coordinated innovation requires broad, sustained collective action
- Such efforts can be organized by a new politics of constructive collective action
- Agricultural scientists must participate actively in these new politics
- Agricultural scientists can become skilled actors in constructive politics of collective action

Another form of collective action is needed to address institutional “lock‐in” mechanisms that support incumbent agricultural socio‐technical systems, making them highly resistant to change (Oliver et al., 2018). These lock‐in mechanisms are manifold, encompassing infrastructure, regulations, public investments such as subsidies and research funding, and cultural attitudes; together, these can strongly inhibit development and scaling of new socio‐technical systems (Meynard et al., 2018). Lock‐in mechanisms must be circumvented to enable new socio‐technical options to emerge. To do so, experience shows that multiple and coordinated interventions must be undertaken at multiple scales (e.g. local, state, national; Meynard et al., 2017). For example, many factors have disincentivized adoption of fallow‐season cover crops in row cropping systems in the United States, effectively locking row‐cropping systems into undesirable losses of soil, nutrients, and water that result from lack of fallow‐season soil cover (Plastina, Liu, Miguez, & Carlson, 2018). Recent gains in adoption of cover crops have occurred because of concerted efforts of many different actors that have begun to transcend this lock‐in: efforts by farmers, small seed companies, and advocates of sustainable farming across the US; a change in federal and state policies; subsidies and technical assistance; actions by major food manufacturers; and scientific reports on the many benefits of cover crops. These efforts are increasingly coordinated by multi‐stakeholder regional groups, e.g., the Midwest Cover Crop Council (http://mccc.msu.edu/). Growing adoption of fallow‐season cover crops shows how collective action can *integrate multiple innovations and interventions* that overcome lock‐in to drive emergence of novel socio‐technical systems.

### 2.3 Premise III: Certain politics are key to broad collective action for innovation and intervention

Evidence suggests that robust collective action on grand challenges and other complex problems depends on a shared vision for addressing the problem, and strategic, adaptive, and tenacious implementation of that vision (Head, Ross, & Bellamy, 2016), and substantial collective investments of intellectual, financial, social, and political capital (Tàbara et al., 2018). We contend that a certain kind of politics can create and sustain the necessary vision, implementation, and investment. We define politics in general as “all societal interactions that address public or common matters” (Levine, 2014). Therefore, the politics of meeting grand challenges are the societal interactions that address these challenges. Because grand challenges are complex problems, these politics are characteristically adversarial (McConnell, 2018), featuring contestation about the nature of the problem, relevant evidence, and potential solutions and allocation of resources thereto (Sumberg, Thompson, & Woodhouse, 2013; Bellwood‐Howard & Ripoll, 2020). Moreover, incumbent governments typically have weak incentives to take decisive action on such problems (McConnell, 2018). These political factors create major barriers to broad collective action on complex problems. Yet, we argue that coherent politics of constructive collective action on such problems are now emerging, and these show much potential to surmount these barriers (Avila, 2017; Boyte, 2011; Dzur, 2018). Certainly, these “constructive” politics will not supersede the adversarial politics of complex problems. Rather, we propose that constructive politics can develop broad coalitions that can make progress on complex problems with histories of adversary politics (Box 1). For this reason, these politics merit close attention from agricultural scientists, and we propose that agricultural scientists can greatly heighten their contributions to meeting these challenges if they understand and participate—as scientists—in these politics. Moreover, to be highly effective participants, they must recognize and embrace a new identity and role: scientist as a skilled *actor in the constructive politics of collective action on grand challenges*, and cultivate certain skills necessary to effective practice of these politics, as highlighted in the model below.

**Box 1: The Forever Green Initiative: Harnessing the Politics of Constructive Collective Action**

The Mid‐Continent of North America is one of the most productive agricultural regions of the world, but cropping systems are dominated by summer annual crops, leaving soil exposed for much of the year and thereby creating a wide range of problems (Asbjornsen et al., 2014). Efforts to address these problems politically have been highly adversarial, pitting environmental interests against mainstream agriculture, with little progress on attaining regional goals, e.g., for water quality. To address this grand challenge, the Forever Green Initiative (https://www.forevergreen.umn.edu/), a public‐private partnership housed at the University of Minnesota, is working to develop and commercialize a set of crops that complement summer‐annual crops in diversified cropping systems. These include intermediate wheatgrass (Kernza®), pennycress, winter camelina, winter barley, and hybrid hazelnut, among others. Each of these crops can enhance continuous living crop cover in the region's agriculture, providing multiple economic and environmental benefits (Asbjornsen et al., 2014; Schulte et al., 2017). The concept of “continuous living cover” by productive crops has proven to be a powerfully unifying concept of “what should be”, attracting bi‐partisan political support, strong private‐sector engagement, and approximately $60 million in competitive funding for crop development and commercialization since 2019. The Initiative has developed a multi‐level structure (Ostrom, 2010), designed to leverage the strengths of “bottom‐up” and “top‐down” strategies for development, via politics of constructive collective action. The structure features a high‐level *Strategic Steering Council* that is supporting deliberative politics, convening public, private, and NGO sectors to define broad strategies for pursuing shared interests in continuous living cover. A set of *pilot projects* practices co‐creative politics, as each project works to improve social, environmental and economic sustainability of regional agriculture by building supply chains for particular Forever Green crops. A third structural element is a mid‐level *Learning/Experimentation Platform* that mediates between the Council and pilot projects. Its activities span deliberative and co‐creative politics, devising and testing means for implementation of the Steering Council's strategies, based on results from pilots. Thus, all of these politics are integral to ongoing innovation and intervention, driven by collective action within and across all levels of the Initiative. The current politics of the Initiative's multi‐level structure leverages social capital created by previous relational politics and network building by its parent organization, the Center for Integrated Natural Resources and Agricultural Management (https://www.cinram.umn.edu).

## 3 A NEW CONSTRUCTIVE POLITICS

We outline a model of constructive politics for broad collective action on grand challenges and other complex problems in agriculture and related systems. These political activities are “constructive” insofar as they support construction of new socio‐technical systems that address such problems. This model features three core elements: relational, deliberative, and co‐creative politics (Levine, 2014). Respectively, these elements describe the tasks of (1) building bonds of affinity within a heterogenous network, (2) developing a roadmap for collective action, and (3) taking sustained action together. Though they are presented individually for sake of clarity, these three elements often operate jointly—in the face of dynamic challenges—as collective action proceeds.

*Relational politics* are the activities needed to build new alliances that enable robust collective action (Dzur, 2018; Levine, 2014). Such alliances are strengthened by interactions that produce mutual understanding and affinity among potential allies. Such understanding and affinity results from inquiry and dialogue to build mutual understanding about the worldviews and capacities of potential partners in collective action (Cooperider & Whitney, 2005). Such relational engagement requires effort, skill, and commitment of time, but is fundamental to effective and sustained collective action. Cognitive flexibility, empathy, and curiosity are needed by all parties. Potential outcomes include discovery of unexpected alignments of interests and underlying values, careful and sympathetic consideration of others’ views and motivations, and recognition of opportunities to exert power through collective action.

We argue that scientists must participate in relational politics to gain the trust of potential partners in broad collective action, especially in an era in which science is increasingly mistrusted (Garlick & Levine, 2017) or deliberately marshalled in adversarial politics (Brown et al., 2010; Oreskes & Conway, 2010). For example, agricultural scientists, as providers and developers of many agricultural innovations, must also understand the motivations and outlook of potential partners in the co‐innovation needed to develop and implement effective new socio‐technical systems for agriculture. Settings for such politics are beginning to emerge that feature agricultural science in integral roles, e.g., multi‐stakeholder platforms to address agricultural problems such as the Southern Africa “Sustainable Agriculture Lab” (Drimie, Hamann, Manderson, & Mlondobozi, 2018), internet‐based coordination (http://globalchangescience.org/eastafricanode/), long‐term multi‐actor innovation systems and learning hubs in South Asia (https://csisa.org/), or “Land Labs” in the Midwest USA (Jordan et al., 2013), among others. Such platforms have inherent costs and challenges, but create institutional settings that enable agricultural scientists to engage in sustained relational politics.

*Deliberative politics* consist of collective learning and deliberation that identifies a shared vision of a desirable future and a feasible pathway to that future (Levine, 2014; Milkoreit, 2017; van Mierlo & Beers, 2018). To build such a shared vision, an alliance formed by relational politics must collectively address the inherent complexity of the grand challenges at hand. This complexity encompasses disparate ideologies, experiences, and stakes among affected people, the histories of the challenge and the places where it is felt, high levels of uncertainty, and institutionalized incentives and other lock‐in factors. Applied to agriculture, deliberative politics requires a sincere, patient, and diligent effort by participants to create and advance a shared image of “what should be” in the agriculture and food systems of the future. Such deliberative politics can nourish and expand cross‐sector alliances that are initially developed by relational politics, by deepening empathy and affinity in the alliance.

In deliberative politics, explicit attention must be given to issues of power and inclusion. It is essential to have dominant actors—which both contribute to and benefit from current food and agricultural systems—“at the table” during such deliberations. Dominant actors can invest their power to advance a shared vision (Bergek, Berggren, Magnusson, & Hobday, 2013) but the alliance must establish and enforce standards of equity and justice (Loos et al., 2014) that prevent dominant actors from coopting the alliance. In particular, less powerful actors must also be at the table, including those most directly harmed by inequity and injustice in food and agricultural systems. Such inclusion respects the ethics of democracy, greatly enhances deliberation by increasing the available range of ideas and experience, and reduces the likelihood that oppositional actors will block the pathway to the alliance's desired future. Deliberation at such tables depends critically on the relational politics described above and will need to grapple with difficult issues of power differentials and representation among participants, sharp tradeoffs between certain interests, and historical legacies of conflict and injustice. Institutions and settings for sustained deliberative politics pose significant implementation challenges, and balancing power among groups and including historically excluded stakeholders are particularly difficult. Yet, emerging understanding of governance by heterogenous networks (Atkins, Wilson, & Hayes, 2019; Ostrom, 2010) provides guidance for design and operation of inclusive deliberative alliances that limit such behavior by dominant actors,

Agricultural scientists have crucial roles in these deliberative politics. First, these scientists can muster empirical evidence to assess the merits of current agricultural systems, and of alternatives that might achieve “what should be”. Also, agricultural scientists can apply their intimate knowledge of how biophysical and social systems work in developing shared visions. Importantly, the discourse of deliberative politics can expand scientists’ imagination of “what should be”, potentially leading to new avenues of discovery and innovation in search of new pathways to collectively preferred futures. If successful, deliberative politics can create an actionable, shared vision of a new socio‐technical system that can forcefully meet a particular grand challenge. This broadly shared understanding provides a map for sustained collective action.

*Co‐creative politics* are the societal interactions that build upon deliberative politics to construct new systems that meet grand challenges. Applied to agriculture's challenges, these politics focus on advancing promising new socio‐technical systems through collective action. Co‐creative politics begin with ongoing, learning‐intensive work to design such action, which is likely to integrate both innovation and interventions, as defined above (Premise II). Of course, defining and implementing coordinated strategies of innovation and intervention is complex and slow. Therefore, ongoing learning is needed so that these strategies are responsive to the dynamic context in which complex problems are understood and framed (Head et al., 2016). Co‐creative politics therefore requires tenacious and adaptive cross‐sectoral communication, dialogue, deliberation, monitoring, and learning. Emerging participatory approaches for holistic evaluation (Grabowski, Musumba, Palm, & Snapp, 2018) can support and sustain such co‐creative processes over time.

Of course, agricultural scientists cannot take on the practice of these relational, deliberative, and co‐creative politics alone. Other sectors, including private sector, government, and advocacy groups, must also be willing and able to engage and share in the inherent costs and risks. In practice, initiatives that aim to bring these politics to bear on agricultural grand challenges (e.g., Box 1) will need a core organizing group of participants attuned to these politics, and that core group must have skilled strategic, facilitation, and coaching support.

## 4 THE WAY FORWARD

We call for exploratory engagement in the constructive politics we outline above. This experiential learning should be coupled to reflective and critical thinking about agricultural science's current modes of engagement with complex problems, potential alternatives to those modes, and the nature and significance of politics in those alternatives.

### 4.1 For individuals

We call on agricultural scientists whose work addresses grand challenges to consider and explore how their work relates to the politics of such challenges. Toward this end, reading, reflection, and conversation with peers can help scientists develop personal conceptions, or “mental models,” of their work in relation to those politics. Key questions include: What role(s) do I, as a scientist, have in societal interactions addressing grand challenges and complex problems? What role(s) does my institution have? How does my research and professional activities affect relevant socio‐technical systems, and how can I engage with those systems? Scientists who choose engagement in these politics will benefit from developing and refining a self‐conception of their politics, including a mental model of their individual and collective political agency (Brown et al., 2010; O'Brien, 2015).

Such self‐reflection lays the personal groundwork for engagement in the politics of constructive collective action. After building a foundation for such engagement, scientists can extend it in low‐risk settings that are nonetheless edifying, such as volunteering with local NGOs or scientific societies. Scientists can also receive training in these politics in a variety of settings (e.g., https://tischcollege.tufts.edu/civic-studies/summer-institute). Undoubtedly, the integration of science and politics is strenuous work, requiring a change of mindset, conviction, and personal growth.

### 4.2 For institutions

We call on positional and thought leaders in agricultural‐science institutions to take stock of how their institutions engage with grand challenges and their associated politics. If these leaders see the need to expand this engagement, then experimentation with institutional incentives, norms, and culture that affect political engagement is called for. For example, building capacity for such engagement in emerging scientists will require investment in learning and practice opportunities for graduate students and early‐career scientists. In our experience, significant numbers of emerging scientists are very eager for such opportunities. In the same vein, faculty and students can be supported in research projects or other scholarly work that is intentionally designed to facilitate constructive collective action as outlined above (Bybee‐Finley & Ryan, 2018). At present, such projects are difficult, due to high transaction costs and limited recognition for political engagement, but strategic institutional investments and reward structures can enable such foundational work. The field of engaged community scholarship has lessons to offer (Sandmann and Jones, 2019), e.g., the University of Minnesota Extension's Regional Sustainable Development Partnerships (https://extension.umn.edu/regional-partnerships). These partnerships are networks that facilitate place‐based, long‐term collective action, providing graduate students and pre‐tenure faculty with many opportunities to engage, as scientists, in constructive collective action and its inherent politics. However, at present, graduate students and pre‐tenure faculty in many countries participate in such activities at considerable risk to their careers. Pro‐active institutional action to reduce that risk is essential.

Beyond the academy, many research, enterprise, administration, and advocacy institutions employ scientists to address grand challenges in agriculture. For scientists working in these institutions, the politics of constructive collective action may offer new leverage in their work. Indeed, “publicly engaged” participatory research approaches (Acevedo, Harvey, & Palis, 2018) are often key to the work of these scientists. We propose that the efficacy of these research approaches can be greatly enhanced by actively embedding them in constructive collective action. Such embedding will require political intentions and skills, as we have outlined above, and relevant training is essential. For this purpose, institutions that employ significant numbers of scientists working on agricultural grand challenges, such as government agencies, NGOs and private firms, can collaborate with academic institutions and others to offer professional development programs on engagement in the politics of collective action. The curricula of such capacity‐building efforts is beyond the scope of this article, but there are many relevant building blocks (Garlick & Levine, 2017).

Finally, we reiterate that agricultural science cannot do these politics alone. To realize the potential of constructive collective action, other participants—e.g., from private, public and civil society sectors—must also see their work as inherently and constructively political, and act accordingly. Universities are a social institution capable of building broad‐based societal capacities for these politics. Therefore, we propose that academic science units should collaborate with other academic units—e.g., in business and law—to jointly build reward structures, institutional support, and training programs that can create extensive societal capacity for these politics.

We close with a conjecture: **engagement in constructive politics of collective action on grand challenges is highly important to sustaining societal support for agricultural science**. We believe that such constructive political engagement will provide an essential foundation to the societal legitimacy, trust, and resource investments that are the lifeblood of our science. Specifically, we propose that if agricultural science is active in cross‐sector innovation and intervention aimed at grand challenges, it will gain increased legitimacy by being seen as working on broadly shared interests, as opposed to those of “interest groups”. It will gain trust through the relational, deliberative, and co‐creative interactions of the politics we have outlined, all of which enable the building of reputation and trust through repeated cycles of dialogue, learning and action (Ostrom, 2003). Robust and sustained investments in agricultural science are more likely if our science is seen as an integral partner in broad‐based societal action to address grand challenges by advancing broad collective. Therefore, we urge agricultural scientists, especially institutional leaders, to actively explore and support participation in the politics of constructive collective action on grand challenges.

## References

[csc220318-bib-0001] Acevedo, M. F. , Harvey, D. R. , & Palis, F. G. (2018). Food security and the environment: Interdisciplinary research to increase productivity while exercising environmental conservation. Global Food Security, 16, 127–132.

[csc220318-bib-0002] Asbjornsen, H. , Hernandez‐Santana, V. , Liebman, M. , Bayala, J. , Chen, J. , Helmers, M. , … Schulte, L. K. (2014). Targeting perennial vegetation in agricultural landscapes for enhancing ecosystem services. Renewable Agriculture and Food Systems, 29, 101–125.

[csc220318-bib-0003] Atkins, P. W. , Wilson, D. S. , & Hayes, S. C. (2019). Prosocial: Using evolutionary science to build productive, Equitable, and Collaborative Groups. Oakland, CA: New Harbinger Publications.

[csc220318-bib-0004] Avila, M. (2017). Transformative civic engagement through community organizing. Sterling, VA: Stylus Publishing, LLC.

[csc220318-bib-0005] Bellwood‐Howard, I. , & Ripoll, S. (2020). Divergent understandings of agroecology in the era of the African Green Revolution. Outlook on Agriculture, *2020*, 0030727020930353.

[csc220318-bib-0006] Bergek, A. , Berggren, C. , Magnusson, T. , & Hobday, M. (2013). Technological discontinuities and the challenge for incumbent firms: Destruction, disruption or creative accumulation? Research Policy, 42, 1210–1224.

[csc220318-bib-0007] Boyte, Harry C. (2011). Constructive politics as public work: Organizing the literature. Political Theory, 39, 630–660.

[csc220318-bib-0008] Brown, V. , Gutknecht, J. , Harden, L. , Harrison, C. , Hively, D. , Jogensen, C. , … Mangel, M. (2010). Understanding and engaging values in policy relevant science. Bulletin of the British Ecological Society, 41, 46–48.

[csc220318-bib-0009] Bybee‐Finley, K. A. , & Ryan, M. R. (2018). Advancing intercropping research and practices in industrialized agricultural landscapes. Agriculture, 8, 80.

[csc220318-bib-0010] Chen, H. G. , & Zhang, Y. H. P. (2015). New biorefineries and sustainable agriculture: Increased food, biofuels, and ecosystem security. Renewable and Sustainable Energy Reviews, 47, 117–132.

[csc220318-bib-0011] Cleveland, D. A. , Müller, N.M. , Tranovich, A. C. , Mazaroli, D. N. , & Hinson, K. (2014). Local food hubs for alternative food systems: A case study from Santa Barbara County, California. Journal of Rural Studies, 35, 26–36.

[csc220318-bib-0012] Cooperider, D. L. , & Whitney, D. (2005). Appreciative inquiry: A positive revolution in change. New York, NY: Berrett‐Koehler.

[csc220318-bib-0013] Delgado, J. A. , Short, N. M. Jr. , Roberts, D. P. , & Vandenberg, B. (2019). Big data analysis for sustainable agriculture on geospatial cloud framework. Frontiers in Sustainable Food Systems, 3, 54.

[csc220318-bib-0014] Dorsch, M. J. , & Flachsland, C. (2017). A polycentric approach to global climate governance. Global Environmental Politics, 17, 45–64.

[csc220318-bib-0015] Drimie, S. , Hamann, R. , Manderson, A. , & Mlondobozi, N. (2018). Creating transformative spaces for dialogue and action: Reflecting on the experience of the Southern Africa Food Lab. Ecology and Society, 23, 2.

[csc220318-bib-0016] Dzur, A. W. (2018). Democracy inside: Participatory innovation in unlikely places. USA: Oxford University Press.

[csc220318-bib-0017] Evans, J. A. , Williams, A. , Hager, A. G. , Mirsky, S. B. , Tranel, P. J. , & Davis, A. S. (2018). Confronting herbicide resistance with cooperative management. Pest Management Science, 74, 2424–2431. 29862629 10.1002/ps.5105 PMC6220798

[csc220318-bib-0018] Garlick, J. A. , & Levine, P. (2017). Where civics meets science: Building science for the public good through civic science. Oral Diseases, 23, 692–696. 27388138 10.1111/odi.12534

[csc220318-bib-0019] Glover, J. D. , Reganold, J. P. , Bell, L. W. , Borevitz, J. , Brummer, E. C. , Buckler, E. S. , … DeHaan, L. R. (2010). Increased food and ecosystem security via perennial grains. Science, 328, 1638–1639. 20576874 10.1126/science.1188761

[csc220318-bib-0020] Grabowski, P. , Musumba, M. , Palm, C. , & Snapp, S. (2018). Sustainable agricultural intensification and measuring the immeasurable: Do we have a choice? In S. Bell & S. Morse (Eds.), Routledge handbook of sustainability indicators and indices (pp. 453–476). Oxfordshire, UK: Taylor and Francis Press.

[csc220318-bib-0021] Head, B. , Ross, H. , & Bellamy, J. (2016). Managing wicked natural resource problems: The collaborative challenge at regional scales in Australia. Landscape and Urban Planning, 154, 81–92.

[csc220318-bib-0022] Herrero, M. , Thornton, P. K. , Mason‐D'Croz, D. , Palmer, J. , Benton, T. G. , Bodirsky, B. L. , … Pradhan, P. (2020). Innovation can accelerate the transition towards a sustainable food system. Nature Food, 1, 266–272.

[csc220318-bib-0023] Jordan, N. , Schulte, L. A. , Williams, C. , Mulla, D. , Pitt, D. , Slotterback, C. S. , … Rickenbach, M. (2013). Landlabs: An integrated approach to creating agricultural enterprises that meet the triple bottom line. Journal of Higher Education Outreach and Engagement, 17, 175.

[csc220318-bib-0024] Kuzma, J. (2019). Procedurally robust risk assessment framework for novel genetically engineered organisms and gene drives. Regulation & Governance, 2019. 10.1111/rego.12245

[csc220318-bib-0025] Leeuwis, C. , & Aarts, N. (2011). Rethinking communication in innovation processes: Creating space for change in complex systems. Journal of Agricultural Education and Extension, 17, 21–36.

[csc220318-bib-0026] Levin, K. , Cashore, B. , Bernstein, S. , & Auld, G. (2012). Overcoming the tragedy of super wicked problems: Constraining our future selves to ameliorate global climate change. Policy Sciences, 45, 123–152.

[csc220318-bib-0027] Levine, P. (2014). Six varieties of politics. P. Levine. Retrieved from http://peterlevine.ws/?p=14549

[csc220318-bib-0028] Loos, J. , Abson, D. J. , Chappell, M. J. , Hanspach, J. , Mikulcak, F. , Tichit, M. , & Fischer, J. (2014). Putting meaning back into “sustainable intensification”. Frontiers in Ecology and the Environment, 12, 356–361.

[csc220318-bib-0029] Ma, X. , Mau, M. , & Sharbel, T. F. (2018). Genome editing for global food security. Trends in Biotechnology, 36, 123–127. 28893405 10.1016/j.tibtech.2017.08.004

[csc220318-bib-0030] McConnell, A. (2018). Rethinking wicked problems as political problems and policy problems. Policy and Politics, 46, 165–180.

[csc220318-bib-0031] Meynard, J. M. , Charrier, F. , Le Bail, M. , Magrini, M. , Charlier, A. , & Messéan, A. (2018). Socio‐technical lock‐in hinders crop diversification in France. Agronomy for Sustainable Development, 38, 54–66.

[csc220318-bib-0032] Meynard, J. M. , Jeuffroy, M.H. , Le Bail, M. , Lefèvre, A. , Magrini, M. B. , & Michon, C. (2017). Designing coupled innovations for the sustainability transition of agrifood systems. Agricultural Systems, 157, 330–339.

[csc220318-bib-0033] Milkoreit, M. (2017). Imaginary politics: Climate change and making the future. Elementa Science of Anthropocene, 5, 62.

[csc220318-bib-0034] O'Brien, K. (2015). Political agency: The key to tackling climate change. Science, 350, 1170–1171. 26785460 10.1126/science.aad0267

[csc220318-bib-0035] Oliver, T. H. , Boyd, E. , Balcombe, K. , Benton, T. G. , Bullock, J. M. , Donovan, D. , … Nunes, R. J. (2018). Overcoming undesirable resilience in the global food system. Global Sustainability, 1, 1–9. 10.1017/sus.2018.9

[csc220318-bib-0036] Oreskes, N. , & Conway, E. M. (2010). Merchants of doubt: How a handful of scientists obscured the truth on issues from tobacco smoke to global warming. New York, NY: Bloomsbury Press.

[csc220318-bib-0037] Ostrom, E. (2003). Toward a behavioral theory linking trust, reciprocity, and reputation In E. Ostrom & J. Walker (Eds.), Trust and reciprocity: Interdisciplinary lessons for experimental research (pp. 19–79). Russell: Sage Foundation.

[csc220318-bib-0038] Ostrom, E. (2010). A multi‐scale approach to coping with climate change and other collective action problems. Solutions, 1, 27–36.

[csc220318-bib-0039] Plastina, A. , Liu, F. , Miguez, F. , & Carlson, S. (2018). Cover crops use in Midwestern US agriculture: Perceived benefits and net returns. Renewable Agriculture and Food Systems, 35, 1–11. 10.1017/s1742170518000194

[csc220318-bib-0040] Rockström, J. , Williams, J. , Daily, G. , Noble, A. , Matthews, N. , Gordon, L. , … de Fraiture, C. (2017). Sustainable intensification of agriculture for human prosperity and global sustainability. Ambio, 46, 4–17. 27405653 10.1007/s13280-016-0793-6 PMC5226894

[csc220318-bib-0041] Sandmann, L. R. , & Jones, D. O. (Eds.). (2019). Building the field of higher education engagement: Foundational ideas and future directions. Sterling, VA: Stylus Publishing, LLC.

[csc220318-bib-0042] Schulte, L. A. , Niemi, J. , Helmers, M. J. , Liebman, M. , Arbuckle, J. G. , James, D. E. , … Witte, C. (2017). Prairie strips improve biodiversity and the delivery of multiple ecosystem services from corn–soybean croplands. Proceedings of the National Academy of Sciences, 114, 11247–11252. 10.1073/pnas.1620229114 PMC5651729 28973922

[csc220318-bib-0043] Steiner, A. , Aguilar, G. , Bomba, K. , Bonilla, J. P. , Campbell, A. , Echeverria, R. , … Zebiak, S. (2020). Actions to transform food systems under climate change. Wageningen, Netherlands: CGIAR Research Program on Climate Change, Agriculture and Food Security (CCAFS).

[csc220318-bib-0044] Sumberg, J. , Thompson, J. , & Woodhouse, P. (2013). Why agronomy in the developing world has become contentious. Agriculture and Human Values, 30, 71–83.

[csc220318-bib-0045] Tàbara, J. D. , Frantzeskaki, N. , Hölscher, K. , Pedde, S. , Kok, K. , Lamperti, F. , … Berry, P. (2018). Positive tipping points in a rapidly warming world. Current Opinion in Environmental Sustainability, 31, 120–129.

[csc220318-bib-0046] van Mierlo, B. , & Beers, P. J. (2018). Understanding and governing learning in sustainability transitions: A review. Environmental Innovation and Societal Transitions, 34, 255–269.

[csc220318-bib-0047] Weltzien, E. , & Christinck, A. (2017). Participatory breeding: Developing improved and relevant crop varieties with farmers In Agricultural systems: Agroecology and rural innovations for development (pp. 259–301). Science Direct, Elsevier.

[csc220318-bib-0048] Whelpton, A. , & Ferri, A. (2017). Private capital for working lands conservation – A market development framework. The Conservation Finance Network. Retrieved from https://www.conservationfinancenetwork.org/2017/04/04/report-private-capital-for-working-lands-conservation

[csc220318-bib-0049] Wigboldus, S. , Klerkx, L. , Leeuwis, C. , Schut, M. , Muilerman, S. , & Jochemsen, H. (2016). Systemic perspectives on scaling agricultural innovations: A review. Agronomy for Sustainable Development, 36, 46.

[csc220318-bib-0050] Willett, W. , Rockström, J. , Loken, B. , Springmann, M. , Lang, T. , Vermeulen, S. , … Murray, C. J. L. (2019). Food in the Anthropocene: The EAT‐Lancet Commission on healthy diets from sustainable food systems. Lancet, 393(10170), 447–492. 10.1016/S0140-6736(18)31788-4 30660336

